# Routine radiographic follow-up is not necessary after physeal fractures of the distal tibia in children

**DOI:** 10.1080/17453674.2019.1643632

**Published:** 2019-07-22

**Authors:** Antti Stenroos, Jussi Kosola, Jani Puhakka, Topi Laaksonen, Matti Ahonen, Yrjänä Nietosvaara

**Affiliations:** a Department of Orthopedics and Traumatology, Töölö Hospital, Helsinki University Hospital;; b Department of Pediatric Orthopedics and Traumatology, Helsinki New Children’s Hospital; Finland

## Abstract

Background and purpose — Unnecessary radiographic and clinical follow-ups are common in treatment of pediatric fractures. We hypothesized that follow-up radiographs are unnecessary to monitor union of physeal fractures of the distal tibia.

Patients and methods — All 224 (147 boys) children under 16 years old treated for a physeal fracture of the distal tibia during a 5-year period (2010–14) in Helsinki Children’s Hospital were included in this study. Peterson type II fractures comprised 55% and transitional fractures (Tillaux and Triplane) 20% of all injuries. Fracture displacement and alignment was measured. Type and place of treatment was recorded. Number of follow-up radiographs and outpatient visits was calculated and their clinical significance was assessed.

Results — 109 children had fractures with < 2 mm displacement and no angulation. The other 115 children’s mean fracture displacement was 6 mm (2–28). 54% of all children were treated by casting in situ in the emergency room, 20% with manipulation under anesthesia and 26% with surgery (internal 57, external fixation 2). Median 3 (1–7) follow-up appointments and median 3 (0–6) radiographs were taken. Follow-up radiographs at or before cast removal did not alter treatment in any of the patients. 223 patients’ fractures healed within 4–9 weeks in good alignment (≤ 5° angulation).

Interpretation — Routine radiographic follow-up is unnecessary to monitor alignment and union of physeal fractures of the distal tibia.

Physeal fractures of the distal tibia represent around 5% of all fractures and 15–20% of physeal fractures in children (Peterson and Peterson 1972, Landin 1983). Appropriate treatment depends on fracture type and displacement, as well as on the age of the child (Cummings 2001, Leary et al. 2009). The aim of treatment is fracture union in good alignment without iatrogenic growth plate damage.

Clinical and radiographic follow-up has been traditionally scheduled to monitor healing of physeal fractures of the distal tibia. The purpose of early clinical follow-up is to check that the cast is appropriate and that operative wounds are healing uneventfully. Radiographs have been taken to register fracture alignment and union, position and integrity of hardware, as well as development of a physeal bar or deformity in patients with growth plate damage. The clinical significance of these outpatient appointments and radiographic follow-up is, however, unclear, which has led Perry et al. (2018) to suggest that distal tibia fracture treatment protocols for children are among top clinical effectiveness research questions to date. Unnecessary outpatient visits and radiographs are a substantial economic burden and consume hospitals and families’ resources. We evaluated whether routine follow-up visits in the outpatient clinic before cast removal and radiographs to monitor healing of physeal fractures of distal tibia are necessary.

## Patients and methods

All children under 16 years old treated for a physeal fracture of the distal tibia during a 5-year period between 2010 and 2014 in the Children’s Hospital, Helsinki University Central Hospital were included in this study (n = 224, 147 boys). Patient identification was done from the hospital’s fracture register (KIDS fracture register). Less than 16-year-old residents of Helsinki are either transported directly or referred for treatment in Helsinki Children’s Hospital with very few exceptions. All patients admitted to the Children’s Hospital for fracture treatment are automatically registered in Kid’s Fracture Tool (New Children’s Hospital Helsinki and BCB Medical) in the emergency department. Likewise patients’ data from the operative theater, orthopedic wards, and outpatient clinic are included in the registry.

**Figure 1.**
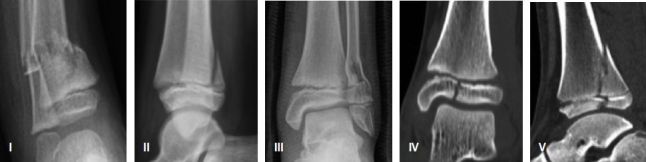
The Peterson fracture classification.

**Figure 2.**
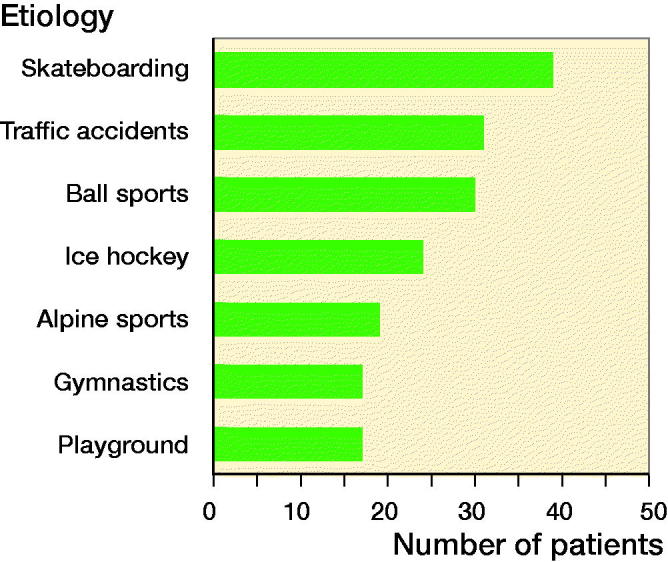
Etiology of distal tibial epiphyseal fractures.

Fracture type (Figure 1) was registered using Peterson classification for patients with open growth plates (Peterson and and Peterson 1972). Transitional fractures (growth plates partially closed at the time of fracture) were divided into Tillaux and Triplane fractures. Etiology of the fractures is presented in Figure 2.

Fracture alignment was assessed from the digital radiographs taken at admission, after reduction, and during follow-ups. Displacement was measured from radiographs as the greatest amount of displacement (mm) in any view. The angle between the shaft and the epiphysis was registered as varus or valgus (AP view), ante- or retrocurvatum (lateral view). Rotational alignment was recorded from patients’ case notes. All patients were followed until fracture union, which was defined as fracture site no longer painful and patient able bear full weight, and radiographic evidence of union (callus on ¾ cortices).

The patients were divided into 3 groups based on their treatment: casting in situ in emergency department (ER), manipulation under anesthesia and casting (MUA), or surgical treatment in operation room (OR). The number of follow-up radiographs and outpatient visits were calculated and their clinical significance was assessed. Cast complications, infections, and reoperations were registered. The targeting, rate, and length of follow-up after fracture union to detect possible growth arrest was evaluated.

### Ethics, funding, and potential conflicts of interest

The ethics committee of Helsinki University Central Hospital approved the study protocol (approval identification number 67/E7/2002). This research received no specific grant from any funding agency. The authors declare no conflicts of interest.

## Results

109 patients had fractures with < 2 mm displacement and no angulation. The remaining 115 patients’ fractures had ≥ 2 mm displacement (median 6 mm, range 2–28 mm) with varus angulation in 98 (median 6°, range 3°–29°) and valgus in 17 (median 7°, range 5°–22°) cases. 121 (54%) patients were treated by casting in ER, 44 (20%) with MUA, and 59 (26%) surgically (internal fixation 57, external fixation 2) (Table 1).

**Table 1.** Basic characteristics of the study population (n = 224) according to different fracture classifications

Factor	Cohort	Cast	MUA	Operative
Peterson:				
I	6 (3%)	3	2	1
II	123 (55%)	80	29	14
III	12 (5%)	6	4	2
IV	21 (9%)	11		10
V	16 (6%)	6	5	5
Tillaux	17 (7%)	7		10
Triplane	29 (13%)	8	4	17
Mean age	12 (1–15)	11 (1–15)	12 (3–15)	13 (8–15)
Leg cast (weeks)	6 (2–8)	6 (2–7)	6 (3–7)	6 (4–8)
Radiographs	3 (0–6)	2 (0–5)	3 (1–5)	2 (2–6)
Follow-ups	3 (1–7)	2 (1–6)	3 (1–6)	3 (1–7)
Time of growth control (months)	7 (3.5–15)	6.5 (3.5–14)	7 (4–13)	8 (4–15)

Values are given as median (range).

**Figure 3.**
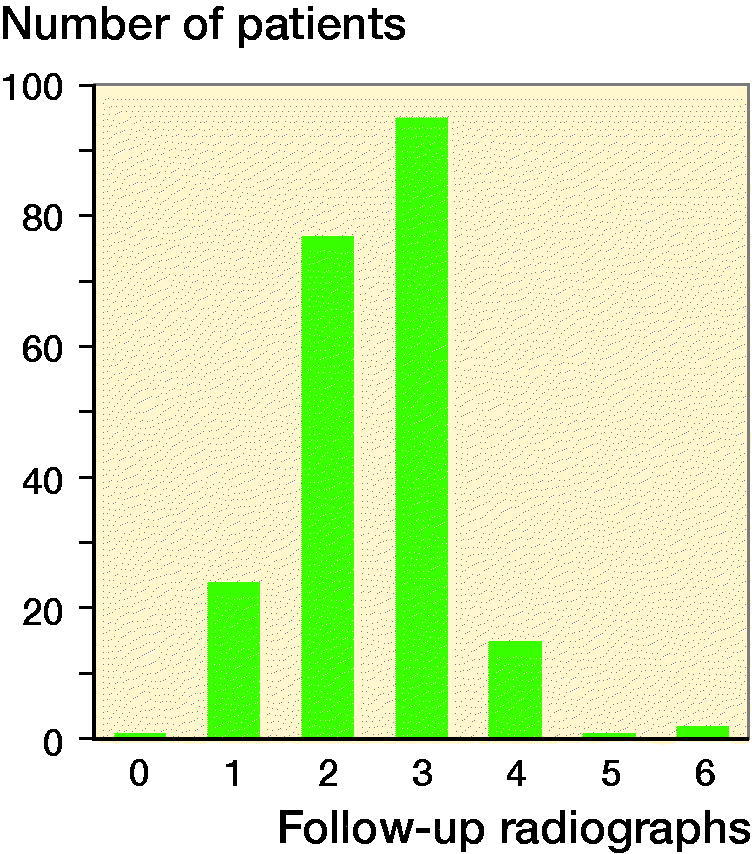
Number of follow-up radiographs to monitor fracture alignment and union of fracture.

The median number of outpatient appointments and radiographs after primary treatment (excluding follow-up to detect possible growth plate damage) was 3 (Figure 3). The first follow-up radiograph was taken at median 10 days (5–52) after the injury in all but one of the patients.

All fractures united at mean 6 weeks (4–9). At the time of fracture union 172 (77%) patients had no, 52 (23%) patients had ≤ 5°, and 1 patient 6° of residual angulation. Follow-up radiographs to monitor alignment and union of the fractures did not alter any patient’s management.

3 reoperations were performed within 48 hours of primary surgery (inadequate reduction 1, hardware in the ankle joint 2). Furthermore 7 patients were treated surgically in our institution after inadequate primary treatment (3 ER, 2 MUA, 2 OR) elsewhere.

17 (8%) patients had cast-related complications (broken cast 7, pain with intact skin 6, full-thickness skin laceration 4). All 4 ulcers were detected at scheduled follow-up appointments (6–9 days from fracture). All these 4 children complained of pain, which led to plaster change during an outpatient visit. 3/59 patients treated surgically developed superficial wound infections that healed with oral antibiotics. 6 patients had additional unplanned surgery 5–33 months after their osteosynthesis (hardware problems 5, tendon release 1).

Further follow-up appointments after fracture union were scheduled for 82 (47%) patients with Peterson type fractures and 13/46 patients with transitional fractures at median 7 months (3–15) from the injury to monitor function of the distal tibial growth plate. The mean primary displacement was higher in the 82 monitored patients vs. 96 non-monitored patients with Peterson type fractures (4.7 mm vs. 2.9 mm). Similar findings were found also concerning primary fracture angulation (4.7° vs. 3.6°). Premature physeal closure was diagnosed in 23/178 patients with Peterson type fractures (13%). 14 of these patients had surgery to correct either angular deformity or leg length discrepancy (Table 2).

**Table 2.** Patients whose growth plate function was monitored according to Peterson classification and method of treatment

Type	Total cohort n = 224	Monitored n = 95	Treatment of monitored		
			Cast n = 40	MUA n = 20	Operative n = 35
Peterson:					
I	6	3	2	–	1
II	123	60	32	14	14
III	12	5	1	2	2
IV	21	7	1	–	6
V	16	7	2	2	3
Tillaux	17	2	1	–	1
Triplane	29	11	1	2	8

## Discussion

To our knowledge, this is the first analysis of the clinical significance of routine follow-up outpatient visits and radiographs to monitor fracture alignment and union of physeal fractures of the distal tibia.

The role of radiographic follow-up in adults’ ankle fractures has been questioned by several authors (Ghattas et al. 2013, McDonald et al. 2014, Ovaska et al. 2016), and it has recently been suggested by Ovaska et al. (2016) that radiographs should not be taken at the first outpatient visit at 2 weeks from primary treatment of adult ankle fractures without clinical signs of a complication. It seems that alignment of physeal fractures of the distal tibia is very unlikely to worsen after adequate primary treatment (Spiegel et al. 1978, Berson et al. 2000, Seel et al. 2011). Our findings support these earlier findings and strongly suggest that routine radiographic follow-up is unnecessary to monitor the alignment of adequately reduced and immobilized/fixed physeal fractures of the distal tibia. We could not find a reason to take a radiograph to document union of these pediatric fractures either, since all 224 fractures in our patients united within 1–2 months in satisfactory alignment.

The reported risk of skin ulcers caused by inadequate casts and surgical wound infection rates after distal tibia fracture has been low (Spiegel et al. 1978, Leary et al. 2009, Seel et al. 2011). We had 4 full-thickness cast ulcers in the 222 patients who had a cast during their treatment, which were all detected during scheduled outpatient visits. This is, however, such a low rate (2%) that routine outpatient visits could perhaps be also abandoned before cast removal. Our findings concerning the proportion of skin problems and the need for cast change should be generalized with caution, because the quality of plaster application probably varies in different institutions. We believe that in our hospital information at the time of discharge on when to return to the emergency or outpatient clinic if potential cast problems arise, such as pain, is adequate.

Our results concerning the rate of growth plate damage should be interpreted with caution, because follow-up was not continued in all patients after union of Peterson type fractures. It was not surprising that our surgeons have not had a uniform scheme to organize follow-up to monitor growth plate function in patients with physeal fractures of the distal tibia, considering there are no distinct guidelines in orthopedic textbooks or publications (Cummings 2001, Herring and Tachdjian 2014, Lovell and Weinstein 2014). Follow-up seems unnecessary for patients with Peterson type I and transitional type (Tillaux, Triplane) fractures, but on the other hand some patients with Peterson type II–V fractures might have benefited from further follow-up after fracture union.

We are going to implement a new written protocol in order to reduce the number of unnecessary follow-ups, which represent a substantial cost to society and patients’ families. (Beiri et al. 2006, Vardy et al. 2014, Holm et al. 2016). All patients with physeal fractures of the distal tibia, irrespective of the method of treatment, are going to have 1 scheduled clinical follow-up at 4–6 weeks from the injury without routine radiographs. Further radiographic follow-up is arranged for all patients with Peterson type II–VI fractures at 6 months from the injury to monitor function of the distal tibial growth plate. Peterson type I fractures and transitional fractures are not routinely monitored after fracture union.

In summary, routine follow-up radiographs to monitor alignment and union of physeal fractures of the distal tibia are unnecessary in patients whose fractures are immobilized, reduced, or fixed in satisfactory alignment. How to best target follow-up to monitor growth plate function in patients with Peterson types II–VI fractures should be assessed. 
